# Post-Treatment Gliosarcoma Extension into the Pterygomaxillary Fossa: Literature Review and Case Report

**DOI:** 10.7759/cureus.700

**Published:** 2016-07-18

**Authors:** Alexander Mason, Alan T Villavicencio, Ewell L Nelson, Robert C Forsythe, Sigita Burneikiene

**Affiliations:** 1 Neurosurgery, Boulder Neurosurgical Associates; 2 Pathology, Boulder Valley Pathology; 3 Neurosurgery, Justin Parker Neurological Institute

**Keywords:** extracranial extravasation, glioblastoma, post-treatment gliosarcoma

## Abstract

Only four primary gliosarcoma case reports are described in the literature with transcranial (intradural to extradural) penetration into the region of the infratemporal fossa. This is the first report of a primary glioblastoma (GBM) that evolved into secondary or post-treatment gliosarcoma without evidence of a second *de novo* tumor and with extension into the left pterygomaxillary fossa.

## Introduction and background

Gliosarcomas are characterized by a typical biphasic tissue pattern composed of gliomatosis and sarcomatous components. Primary gliosarcomas account for approximately 2% of glioblastomas (GBM) [1], but only about 50 cases of secondary or post-treatment gliosarcomas are reported in the literature to date with the largest series including 30 patients [2]. According to Han et al., [3] secondary gliosarcomas and radiation-induced gliosarcomas should be considered separate clinical diagnoses. Radiation-induced gliosarcomas are differentiated in that they occur as lesions within the radiation field, without a prior diagnosis of GBM in patients undergoing radiotherapy for various indications, and they take longer to develop.

To the best of our knowledge, this is the first report of primary GBM that evolved into secondary or post-treatment gliosarcoma without evidence of a second *de novo* tumor and with extension into the left pterygomaxillary fossa. 

## Review

Although due to the prevalence of mesenchymal component, dural invasion and metastasis are more common in gliosarcoma than GBM; only a few cases of post-treatment gliosarcomas with extracranial metastases were reported. Those were predominantly multiple systemic metastases spreading via hematogenous dissemination to the chest, abdomen, and pelvis [4-6] or were found adjacent to the craniotomy site due to iatrogenic tumor cell infiltration into the surgical defect [4, 6-7].

Direct extracranial infiltrations and skull base extensions of either GBMs or gliosarcomas remain a poorly understood and extremely rare phenomenon. This is because tumor invasion may be hindered by the dura mater and tight junctions between the blood vessel endothelial cells, which act as a barrier to malignant cell spread. It was previously believed that the lack of the lymphatic system in the brain may also prevent cancer cell spread [8], but since the discovery of functional lymphatic vessels in the dural sinuses that are connected to the deep cervical lymph nodes, this may no longer be the case [9].

Extracranial GBM extensions to the neck, ethmoidal, maxillary and sphenoid sinuses, orbit, pterygomaxillar and nasal fossa [10-15], parotid gland [16], and retroauricular region [17] have been reported. Only four primary gliosarcoma case reports are described in the literature with transcranial (intradural to extradural) penetration into the region of the infratemporal fossa [18-21]. This is the first report of secondary gliosarcoma with extension into the left pterygomaxillary fossa. 

### Case Report

**Figure 1:**
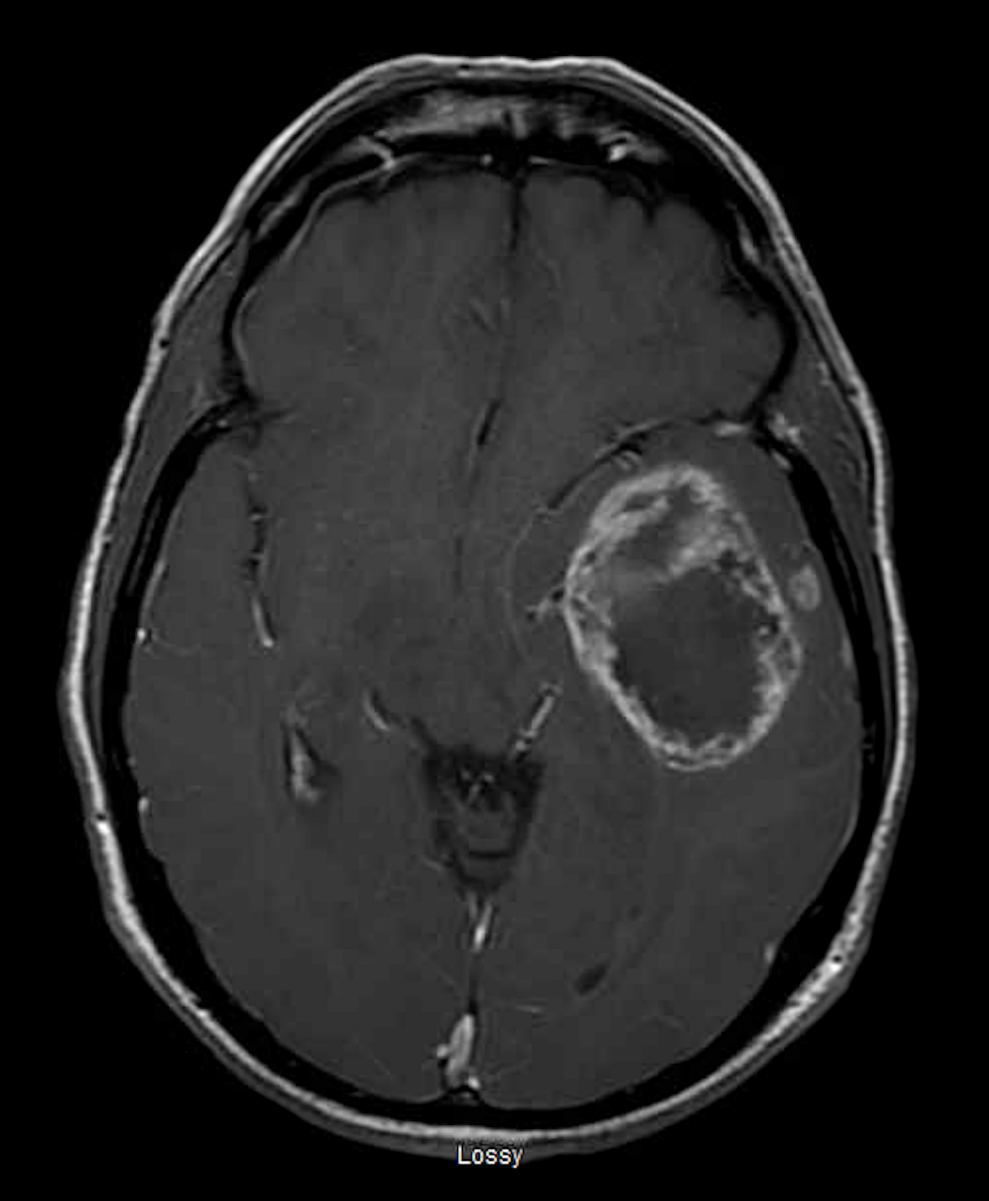
Brain MRI, Axial View with Contrast Enhancement

**Figure 2:**
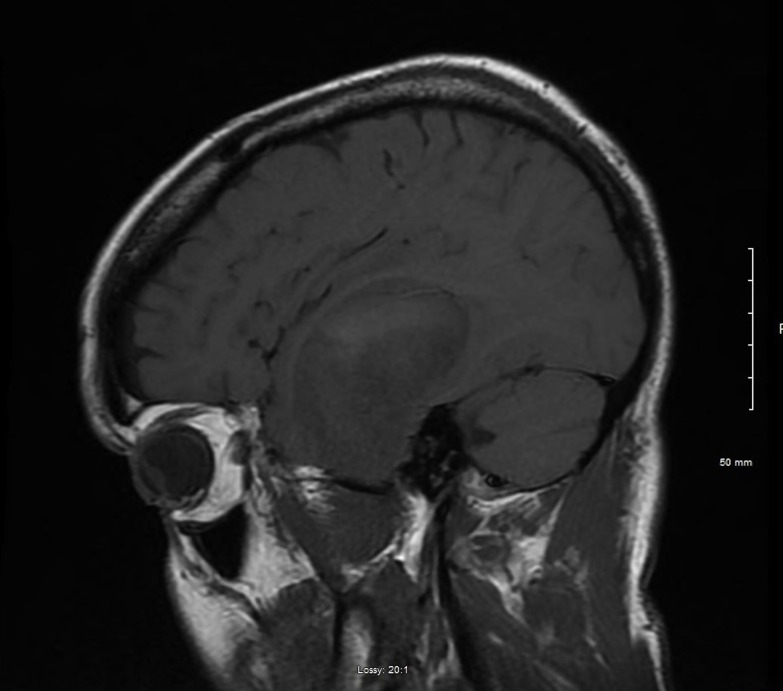
Brain MRI, Sagittal View Without Contrast Enhancement

A 56-year-old male initially presented with gradually worsening and localized, left sided headaches that became so severe he presented to the emergency department. He was found to be mildly aphasic with memory loss symptoms that had been ongoing for a couple of months. The rest of his clinical examination was normal. Brain magnetic resonance imaging (MRI) with (Figure 1, axial view) and without contrast (Figure 2, sagittal view) revealed a large, left temporal intra-axial mass with a maximum diameter of 5.8 cm, associated T2 non-enhancing central necrosis, and vasogenic edema. Two satellite nodules were present: one along the lateral margin (7 mm) and a second along the anterior-inferior margin (6 mm) of the lesion. The lesion compressed the left lateral ventricle and resulted in a 5 mm left-to-right shift. 

**Figure 3:**
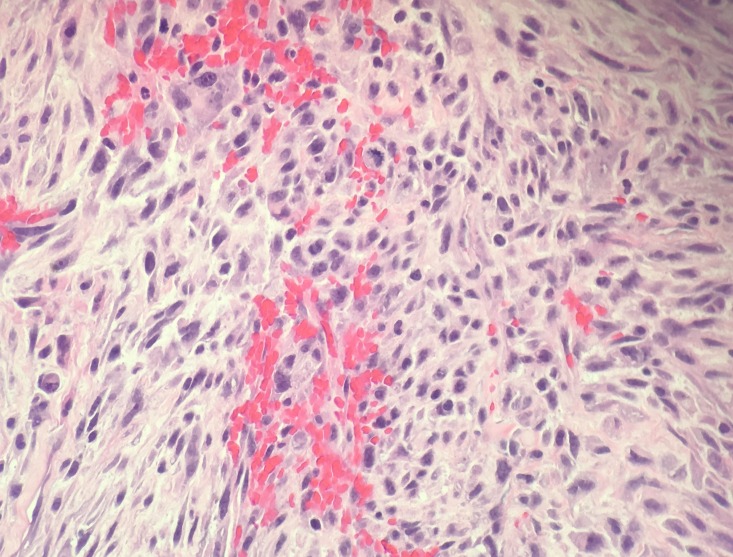
Histology Slide A 400x magnification view of a region of glioblastoma exhibiting hypercellularity, cytologic pleomorphism, intratumoral hemorrhage, and occasional mitotic activity.

A left sided craniotomy and gross total resection of the lesion was performed using computer volumetric, stereotactic navigation and awake speech mapping. Histopathological analysis demonstrated an infiltrating glial neoplasm with occasional microcalcifications, extensive microvascular proliferation, vascular thrombosis, and pseudopalisading necrosis consistent with a diagnosis of GBM (Figure 3). Further, cytogenetic studies found no evidence of epidermal growth factor receptor (EGFR) amplification, but there were phosphatase and tensin homolog (PTEN) deletion and chromosome 10 loss.

**Figure 4:**
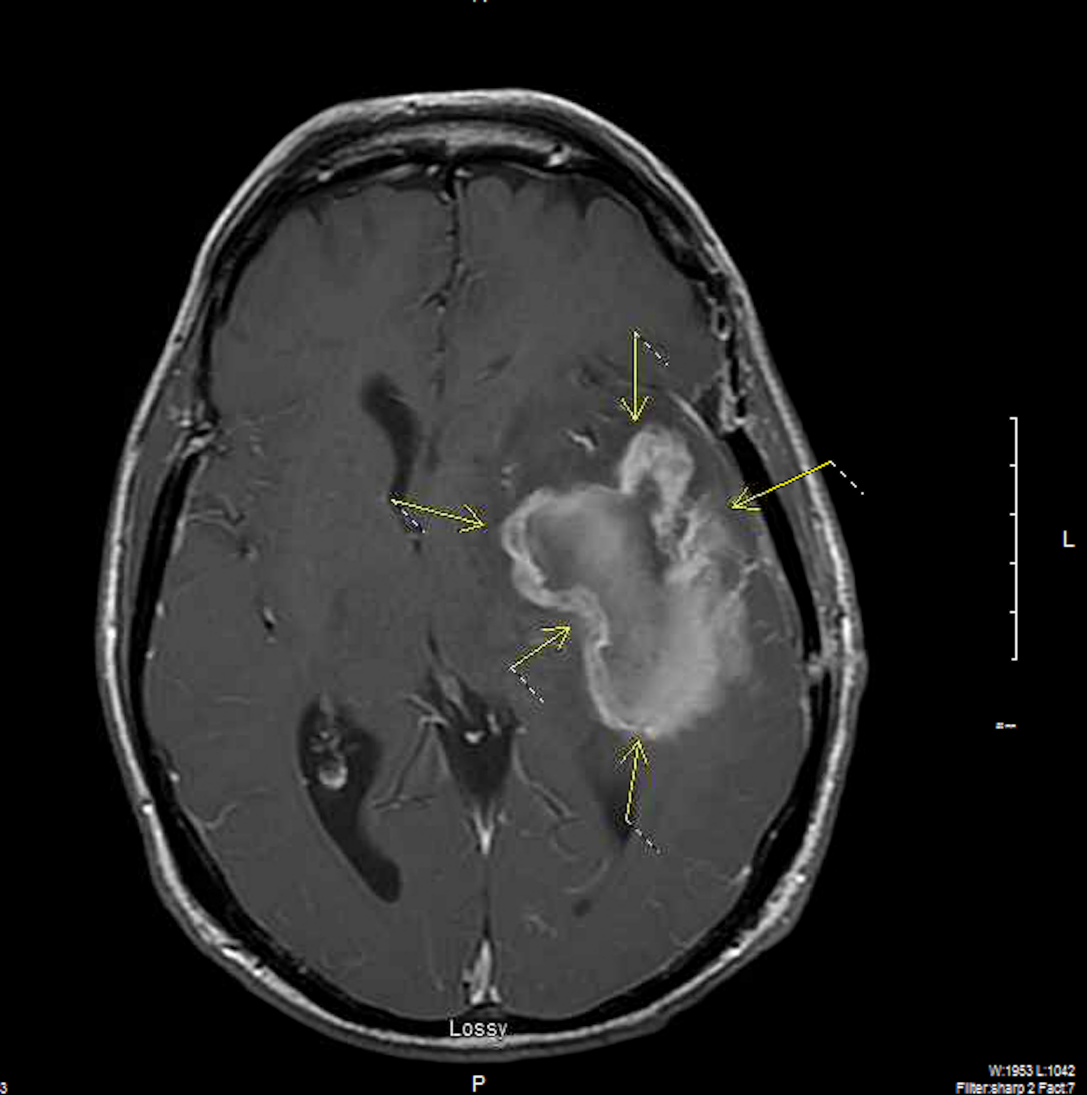
Brain MRI, Axial View with Contrast Enhancement Peripheral enhancement of the left temporal lobe operative bed and associated vasogenic edema consistent with residual or recurrent neoplastic change.

**Figure 5:**
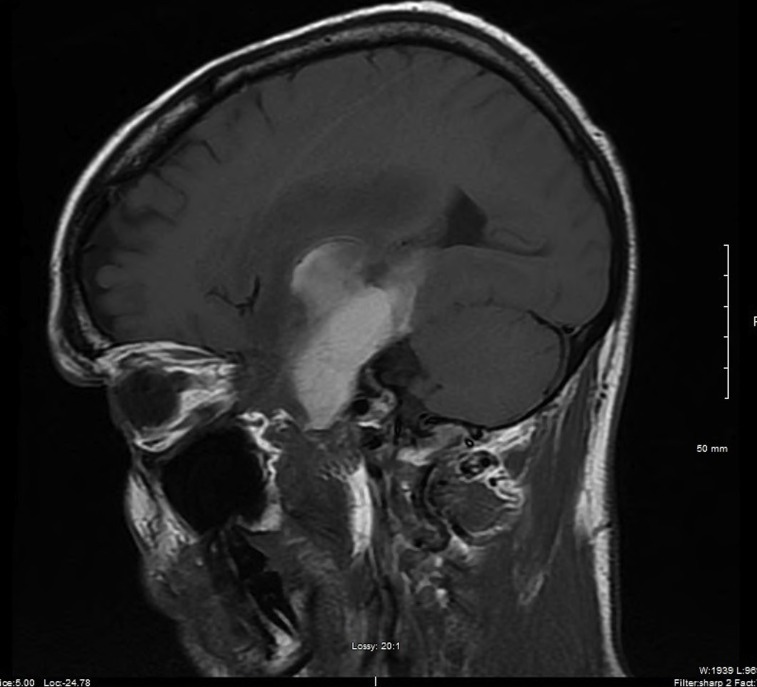
Brain MRI, Sagittal View Without Contrast Enhancement

Less than a month after surgery, the patient presented with worsening aphasia, focal motor seizures, increasing mass effect, and brain MRI showing possible recurrent or residual tumor (Figure 4, axial view with contrast; Figure 5, sagittal view without contrast).

**Figure 6:**
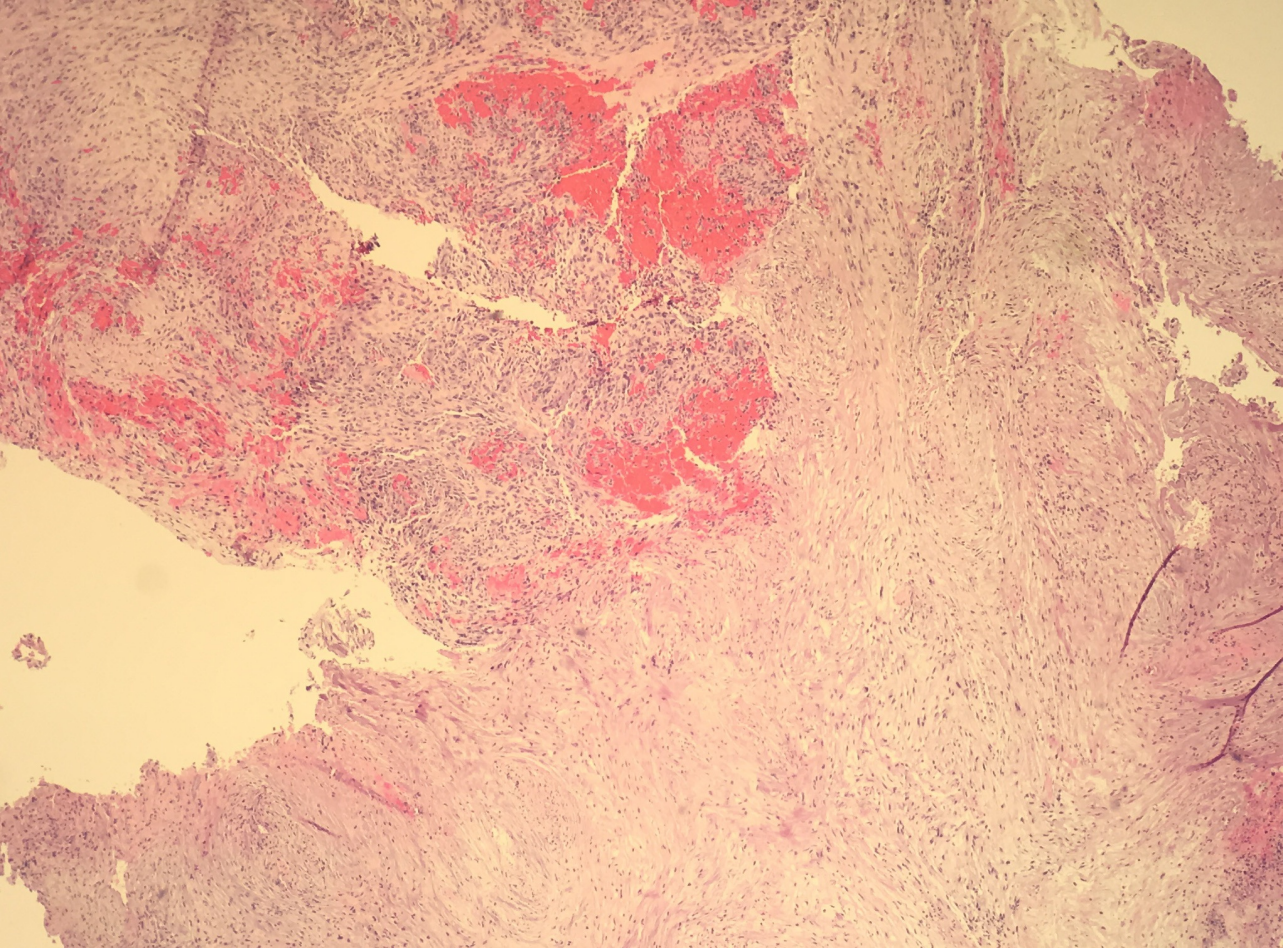
Histology Slide A 40x magnification view showing a region of more traditional glioblastoma in the upper left aspect exhibiting greater hypercellularity and epithelioid cytology. This region shows relatively abrupt transition to a more paucicellular spindle cell proliferation in the lower right, consistent with gliosarcoma.

Re-resection was undertaken to access therapy-induced changes vs. recurrent tumor. A small focus of viable high-grade glioma histologically similar to the original tumor was identified at the edge of reactive tissue (Figure 6).

**Figure 7:**
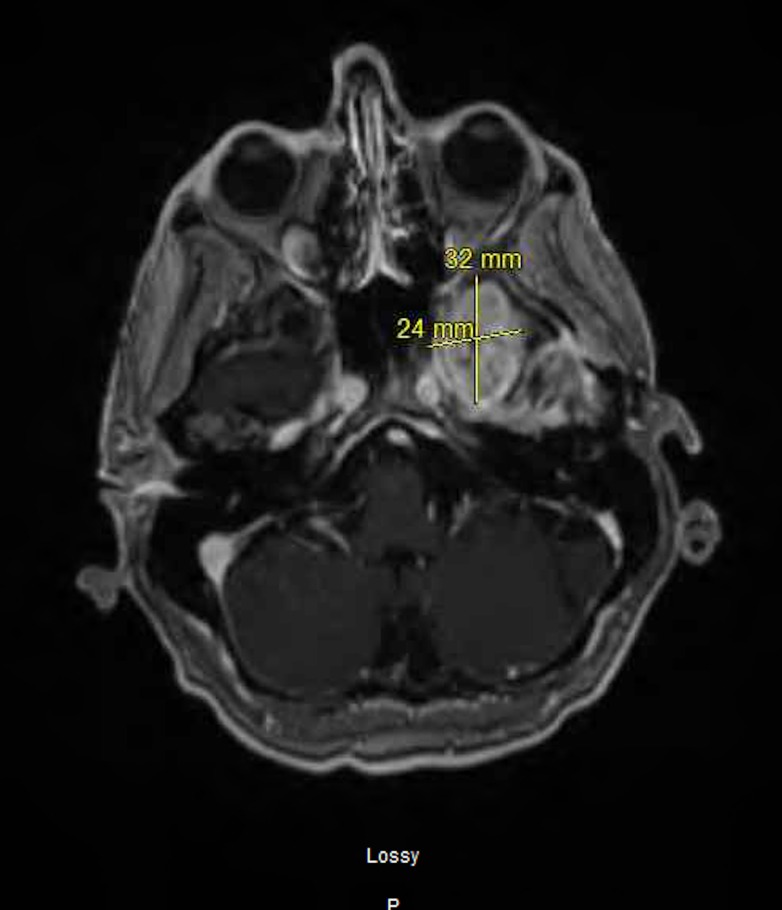
Brain MRI, Axial View with Contrast Enhancement A large temporal lobe mass bridging the skull base into the left pterygomaxillary region.

**Figure 8:**
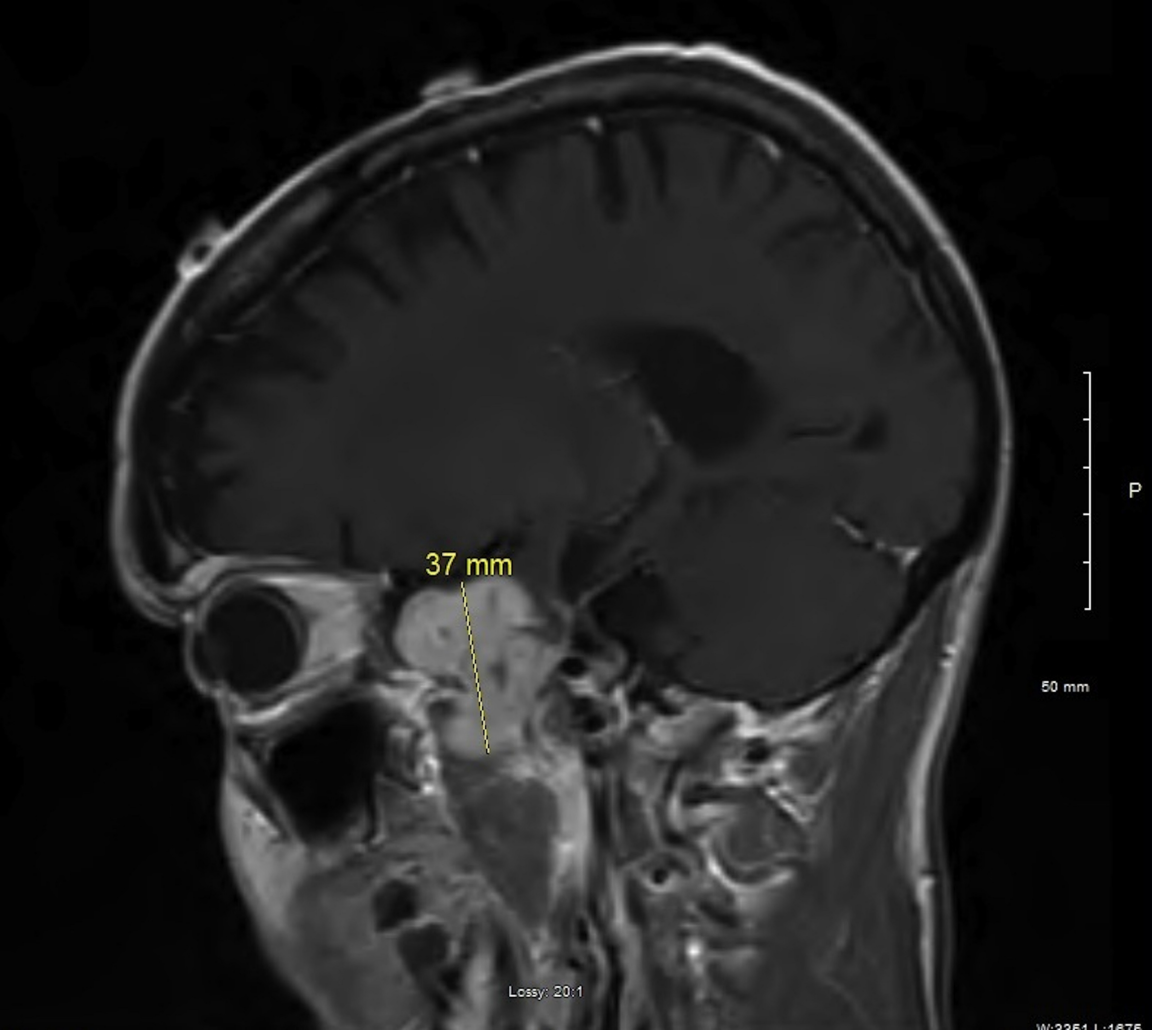
Brain MRI, Sagittal View Without Contrast Enhancement A large temporal lobe mass bridging the skull base into the left pterygomaxillary region.

The patient was managed with external-beam radiation delivered by conventional fractionation (total dose delivered was 54 Gy) and chemotherapy, i.e., daily temozolomide and weekly irinotecan. Subsequently, he received courses of chemotherapy consisting of temozolomide and either carmustine or irinotecan and Taxol. The patient was doing very well with no evidence of progressive disease for 2 years and 8 months after the initial diagnosis when he presented with worsening headaches. Brain MRI was performed (Figure 7, axial view with contrast; Figure 8, sagittal view without contrast). This demonstrated recurrence of the lesion; a large left extra-axial temporal mass was seen on MR images, which extended through the skull base into the left pterygomaxillary region. The mass measured 3.7 cm x 3.2 cm x 2.4 cm. The patient underwent repeat craniotomy with partial resection.

**Figure 9:**
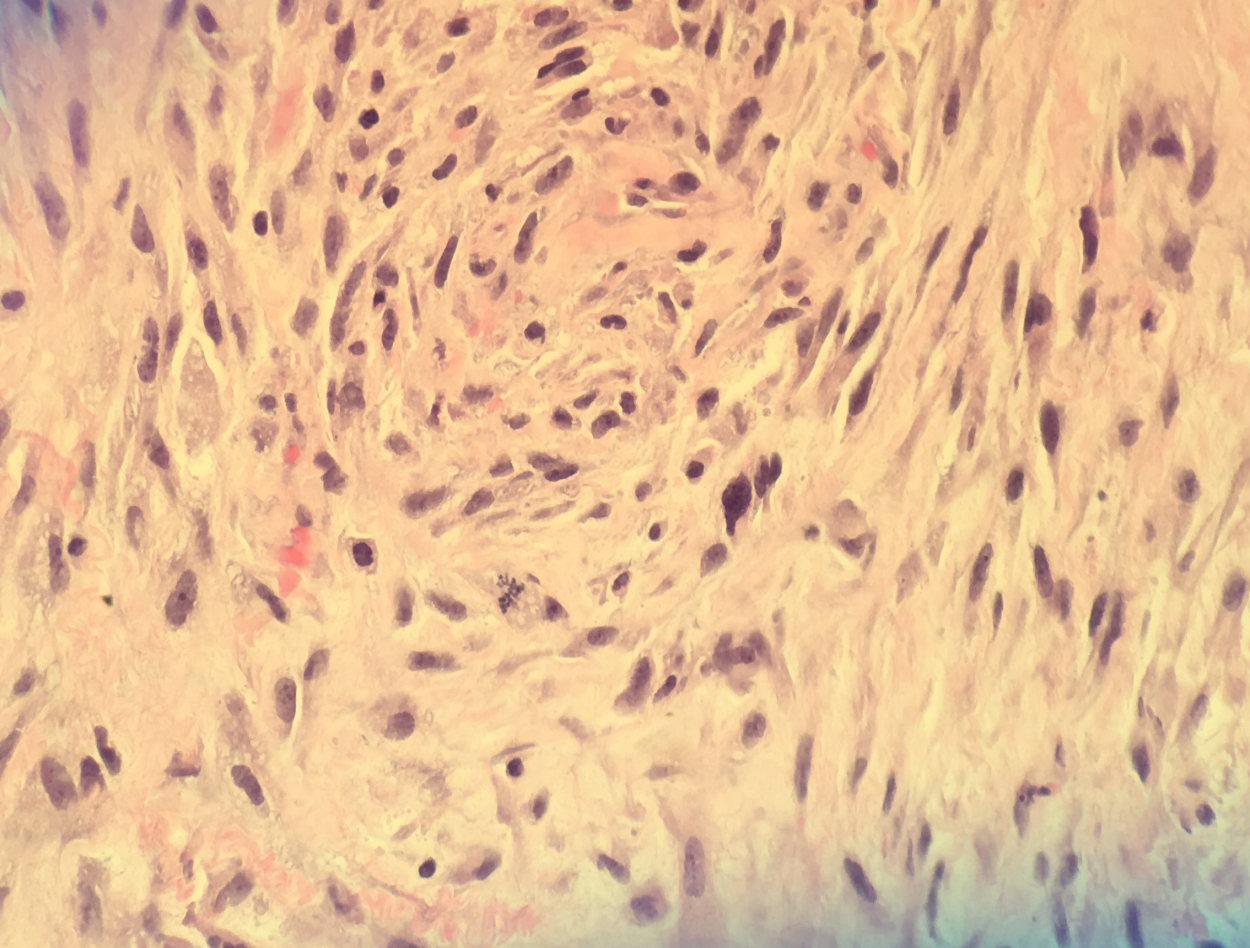
Histology Slide A 400x magnification view of a region of gliosarcoma exhibiting a moderately cellular, atypical spindle cell proliferation containing occasional abnormal mitotic figures (center).

The histopathological analysis demonstrated spindle cell neoplasm ranging from paucicellular, moderately cellular, to high cellularity associated with an elevated mitotic rate. Occasional regions of vascular proliferation and hemorrhage were noted within the tumor. It was classified as gliosarcoma, WHO Grade IV (mixed glioblastoma/sarcoma) (Figure 9). The patient died two months after the surgery.

There is limited clinical experience with post-treatment gliosarcomas presented in the literature. The largest series was reported by Han et al., [2] and included 30 consecutive patients. The median time to gliosarcoma diagnosis was 8.5 months after initial GBM diagnosis. The median length of survival was 4.4 months and 12.6 months from gliosarcoma and GBM diagnoses, respectively. Shorter survival was noted for patients treated with radiation and temozolomide. This was a retrospective study, and molecular or cytogenetic analyses were not performed. The authors hypothesized that these patients may have a unique molecular GBM profile, which could help to distinguish which GBMs can recur as gliosarcomas. They also noted a larger volume of secondary gliosarcomas recently, which could be related to either more aggressive management and prolonged survival of GBM patients or increased awareness of this phenomenon. In the case presented here, the patient was also treated with radiation and temozolomide and the survival time was 32 and 2 months from GBM and gliosarcoma diagnoses, respectively.

According to the Han et al.’s definition [3], the case presented in this report would be considered a secondary or post-treatment gliosarcoma with extracranial extension. The extent to which radiation played a role in this patient’s GBM transformation to gliosarcoma is unclear, although it evolved in the area of prior irradiation almost 3 years after exposure to both radiation and chemotherapy. In addition, the tumor was growing outside the surgical path; therefore, the possibility of iatrogenic tumor cell infiltration should also be excluded.

Gliosarcomas have a similar cytogenetic profile to primary GBM with the exception of epidermal growth factor receptor (EGFR) amplification [22], which is rare in gliosarcomas, while EGFR is overexpressed in about 50% of GBM cases [23]. Although EGFR amplification was not observed in our patient, the histopathological analysis demonstrated two morphological features--necrosis and endothelial proliferation, essential for GBM diagnosis.

It has been reported that post-treatment gliosarcomas have a greater potential to metastasize compared to primary gliosarcomas [6], but they rarely destroy the adjacent bone and extend into the underlying skull base. We are not currently aware of any cases reporting post-treatment gliosarcoma with extracranial extension. There are reports of this behavior in a few primary gliosarcoma cases [18-21], but this has not yet been reported among post-treatment gliosarcomas. Due to the rarity of gliosarcomas, unclear mechanisms of pathogenesis, and limited clinical experience, clinical management of these tumors is challenging. Similar to the treatment of GBM, an extensive multimodality approach is recommended for treatment of primary gliosarcomas. Sade et al., [20] reported a 6 cm primary temporal gliosarcoma tumor spreading into the infratemporal fossa and the posterolateral sphenoid sinus. Gross total resection of the tumor was performed, and the patient was recurrence-free at the 12-month follow-up evaluation after undergoing treatment with fractionated whole brain radiotherapy and temozolomide. The same management recommendations may not apply to the patients with post-treatment gliosarcomas because the possibilities are limited after the primary GBM treatment and prognosis is dismal. A median survival of 4.4 months (range, 0.7 – 46) was reported for a total of 30 post-treatment gliosarcoma patients [2], and gross total resection was performed in 21 (70%) patients. Chemotherapy with different agents was provided for 14 (47%) patients, and only 5 (17%) patients received either external beam radiation therapy or gamma knife surgery. Due to the highly invasive nature of extracranial extensions, only partial resection was possible in this case as the tumor invaded the bone including along the cavernous sinuses.

## Conclusions

This is the first report of primary GBM that evolved into secondary or post-treatment gliosarcoma without evidence of a second *de novo* tumor and with extension into the left pterygomaxillary fossa. There are a few cases of primary gliosarcomas with extracranial extension, but this was not reported among post-treatment gliosarcomas. Due to the rarity of gliosarcomas, unclear mechanisms of pathogenesis, and limited clinical experience; clinical management of these tumors is challenging. In the case presented here, the survival time was 32 and 2 months from GBM and gliosarcoma diagnoses, respectively. 
